# Unusual Occurrence of Tongue Sensorial Disorder after Conservative Surgical Treatment of Lymphoepithelial Cyst

**DOI:** 10.1155/2015/352463

**Published:** 2015-05-11

**Authors:** Luane Macêdo de Sousa, Assis Felipe Medeiros Albuquerque, Paulo Goberlânio Barros Silva, Thâmara Manoela Marinho Bezerra, Ealber Carvalho Macedo Luna, Filipe Nobre Chaves, Francisco Samuel Rodrigues Carvalho, Karuza Maria Alves Pereira, Ana Paula Negreiros Nunes Alves, Thyciana Rodrigues Ribeiro, Fábio Wildson Gurgel Costa

**Affiliations:** ^1^Department of Morphology, Federal University of Ceará, Rua Delmiro de Farias, s/n, 60416-030 Fortaleza, CE, Brazil; ^2^Department of Clinical Dentistry, Federal University of Ceará, Rua Alexandre Baraúna 949, 60430-160 Fortaleza, CE, Brazil; ^3^Division of Oral Pathology and Stomatology, Federal University of Ceará, Campus Sobral, Rua Coronel Estanislau Frota, s/n, 62010-560 Sobral, CE, Brazil; ^4^Department of Oral and Maxillofacial Surgery, Walter Cantídio University Hospital, Federal University of Ceará, Rua Alexandre Baraúna 949, 60430-160 Fortaleza, CE, Brazil

## Abstract

Lymphoepithelial cyst is a rare lesion of the oral cavity, with the mouth floor being the most common site of occurrence. The therapeutic approach of choice is the surgical treatment, which has rare cases of postoperative complications. The aim of this study is to report the case of a 53-year-old patient who came to Dental Service in the Federal University of Ceará complaining of a small nodular lesion (0.5 cm) located in the ventral tongue. Excisional biopsy was performed and the surgical specimen was submitted for anatomopathological analysis, which found that there was an oral lymphoepithelial cyst. The patient returned after seven days for suture removal and reported loss of sensitivity around the ventral tongue. We prescribed Citoneurin for ten days; however, there was not any significant improvement of the sensitivity. Low frequency laser therapy sessions were applied. The only postoperative symptom was dysesthesia, where there is only a sensitivity decrease. Currently, the patient has a postoperative period of 1 year without recurrence of the lesion. Although previous reports have no described tongue sensorineural disorders associated with this lesion, the occurrence of this event may be related to an unexpected anatomical variation of the lingual nerve.

## 1. Introduction

The lymphoepithelial cyst (LC) is a rare lesion of the oral cavity [1–6]. The mouth floor is the most affected site, followed by the lateral and ventral margin of the tongue [1, 3–5, 7, 8]. Some cysts may also occur in the soft and hard palate, retromolar area, palatoglossus arch, palatine tonsil, jaw, and parotid gland, which may indicate, in this last case, an early infection by HIV [4, 5, 9–11].

The LC can occur at any age, affecting mainly adults in the second and third decade of life, with a slight predilection for male gender [6, 9, 12]. Its etiology was not yet well understood, with local trauma being the main hypothesis [1, 13]. Another suspected cause for LCs is the obstruction of a crypt of an oral tonsil, which can be caused by the accumulation of squamous epithelial cells and/or purulent materials [3, 4, 6, 14].

Clinically, the LC appears as a small submucous swelling usually soft to palpation, with color ranging from pink to yellow papule and, at several times, containing keratin, which leads to a creamy or cheesy appearance [4, 6, 8, 12, 13]. Generally, it is asymptomatic [4, 8, 13], but the pain can occur after a secondary trauma [6, 14]. The lesion usually is not larger than 1.5 cm in diameter and, due to the small size and the absence of symptoms, it is only diagnosed after detailed examination of the oral cavity [6–8, 12]. The histopathological exam shows a cystic cavity with a variable amount of desquamated epithelial cells lined by stratified squamous epithelium predominantly parakeratinized of planar interface with conjunctive tissue [4, 9, 12]. The fibrous capsule displays a dense lymphocytic infiltrate, with the presence or absence of germinative centers [5, 6].

Treatment for oral LC has usually been done by conservative surgical excision, with rare cases of recurrence [4–6, 12, 13]. Although surgical procedures in tongue have been related to nerve injury in this region, there are no reports describing the occurrence of sensorineural disorder in the tongue of patients undergoing excision of LC. Thus, the objective of this study was to report an unusual case of paresthesia in the ventral surface of the tongue related to the removal of LC.

## 2. Case Report

**Figure 1:**
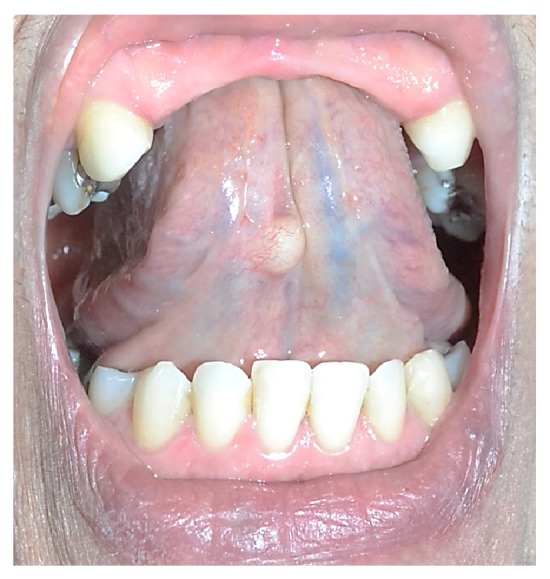
Clinical aspect of the lesion in right ventral tongue region.

Female patient, pale skin, 53 years old, was evaluated at the dental service for prosthetic rehabilitation. After a detailed examination of the oral cavity, the presence of a nodular yellowish lesion with a smooth surface, sessile base, well-defined contours, located in ventral surface of the tongue, right side, measuring 0.5 cm, was confirmed (Figure 1). The patient did not have upper anterior and lower posterior teeth. She reported the presence of this “bubble” for about 1 year ago, but denied soreness, local trauma history, secretion drainage, or lesion growth.

With the diagnostic hypothesis of oral LC, an excisional biopsy was performed under local anesthesia with mepivacaine 2% (1 : 100,000 epinephrine). An elliptical shape incision was performed, superficial and tangent to the lesion base. Next, there was blunt dilatation, hemostasis review and synthesis with silk thread 4.0. Formaldehyde 10% fixed the surgical specimen, which then was sent to anatomopathological analysis.

**Figure 2:**
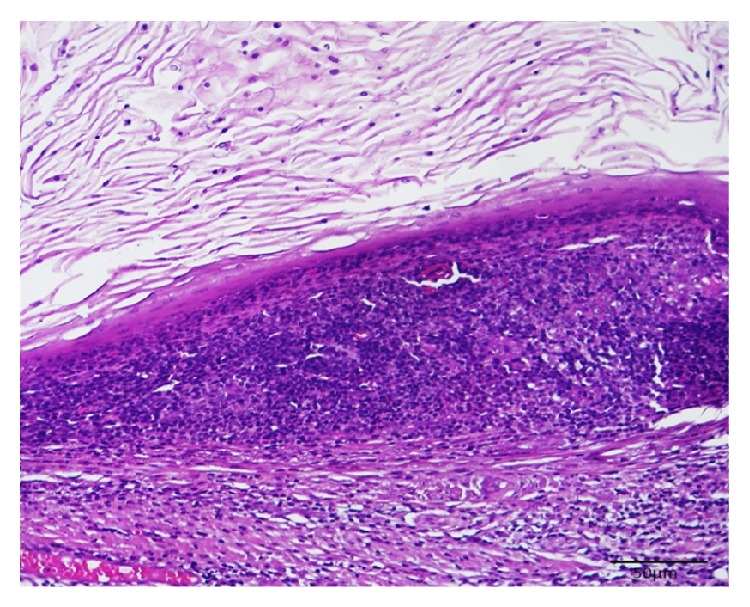
Histological section stained with hematoxylin-eosin, showing a stratified squamous epithelium predominantly parakeratinized, flat interface with the conjunctive tissue, showing a dense lymphocytic infiltrate (HE ×100).

**Figure 3:**
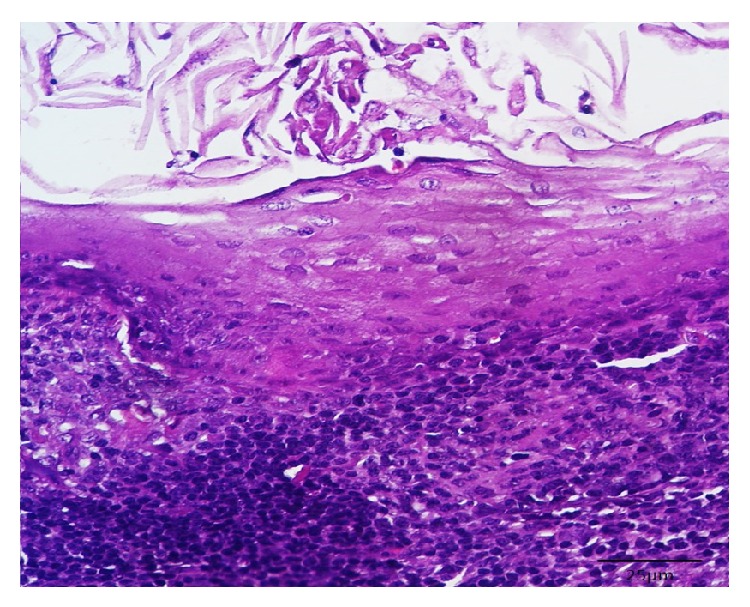
Detail showing the largest increase in fibrous capsule and a dense lymphocytic infiltrate (HE ×400).

Histological sections stained with hematoxylin-eosin showed a cystic cavity containing a variable amount of squamous epithelial cells covered with stratified squamous epithelium predominantly parakeratinized, presenting flat interface with the conjunctive tissue (Figure 2). The fibrous capsule showed a dense lymphocytic infiltrate (Figure 3), without identification of germinal centers, compatible with oral LC.

**Figure 4:**
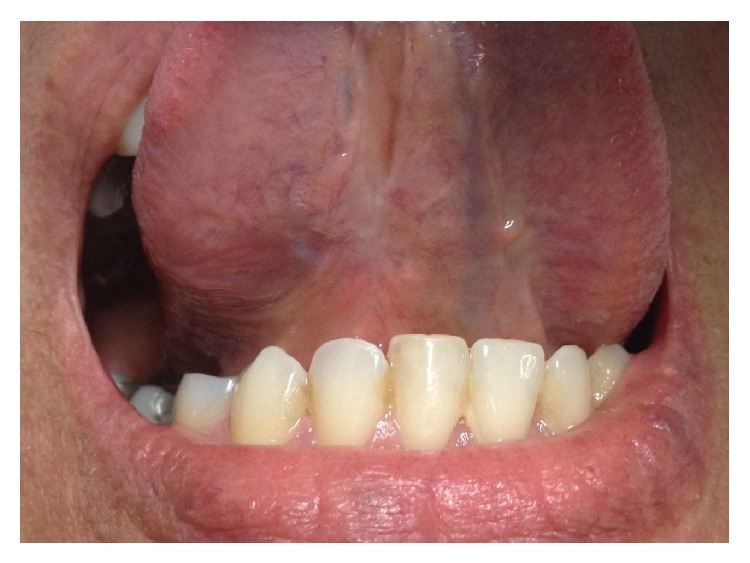
Postoperative of 6 months showing no clinical signs of recurrence of the lesion.

The patient returned after seven days for suture removal and reported loss of sensation in the region around the ventral surface of the tongue. Citoneurin (Merck, São Paulo, Brazil) every twelve hours for ten days was prescribed. During the return visit after this period of medication, the patient did not report significant improvement in sensitivity. Then, low-intensity laser therapy sessions with the Photon Laser III device were done (DMC Equipment LTDA, Brazil). The established protocol for this case was as follows: two applications per week of infrared laser diode (808 nm frequency, dose of 100 J/cm^2^, 28 seconds per application), interleaved by one day for a month. Before each application, the paresthesia region was bordered through mechanical testing using a gingival needle. After the laser therapy, the patient reported a partial recovery of sensibility, which was located only in the surgical procedure area. Using the pain sensation test with gingival needle, there was a decrease in sensitivity at the biopsy site, characterizing this postoperative numbness in dysesthesia. The patient has a postoperative period of 1 year without recurrence of the lesion (Figure 4).

## 3. Discussion

Lymphoepithelial cyst is a benign lesion, very rare in the oral cavity, representing between 0.09% and 0.18% of all lesions diagnosed in Oral Pathology Services [6, 12, 14]. The most prevalent site is the mouth floor region (70.7%), followed by lateral margin (10.7%) and ventral surface (7.3%) of the tongue [7]. The reported case, located in the ventral surface of the tongue, corroborates with the literature.

As noted in the present case, the LCs usually appear solitary in the oral cavity, but there are reports of intraosseous bilateral LCs in jaw and palatine tonsils, and multiple cysts in the parotid gland region in patients affected by HIV, which is one of the signs of the virus infection [9–11]. Our group previously reported an unusual case of multiple LCs located in sublingual region in a patient without systemic involvement [15].

The oral LC occurs in a wide range of age, and most of the cases are diagnosed between the second and third decades of life, with slight preference for male patients [6, 12]. About the preferential involvement in men, and in this age range, the present case was diagnosed in a woman in the fifth decade of life, not corroborating with the literature findings. In contrast, Yang et al. [16] conducted an analysis of 120 cases of LCs treated from 1993 to 2010 in the Oral and Maxillofacial Surgery Department, with 37 being men and 83 women, with a proportion man: woman of 1 : 2.24. The fourth decade of life was the most affected age group.

Histopathologically, the oral LC reveals a cystic cavity lined or partially lined by a stratified squamous epithelium with desquamated keratin in the lumen [4]. The cystic lumen demonstrates a flat epithelial-conjunctive interface, displaying varying amounts of squamous epithelial cells and inflammatory cells [14]. In the present case, the cystic cavity was lined by epithelial, which predominated the pattern of the parakeratinized cell maturation. The fibrous capsule contains an intense lymphocytic infiltrate, and germinal centers are frequently observed [6, 14]. Contrary to the case reported, formation of germinal centers was not identified.

Regarding differential diagnosis, several lesions should be evaluated such as mucocele, lipoma, fibroma, sialolithiasis, submandibular gland cyst, and dermoid cyst [14, 15]. In mucocele cases, the lesion may present a clinical history of growth, followed by rupture, and further growth, due to mucin accumulation. Fibromas and lipomas are located deeper in the mucosa and feature a flat texture [8, 9]. These are mostly found in oral mucosa [8]. The sialolithiasis and the sublingual gland cyst are more easily differentiated from oral LC, except for lesions with small size [8, 9]. The dermoid cyst is located usually on the midline of the oral cavity floor and features a rubbery consistency [8]. In this case, the lesion characteristics mainly suggested beyond the LC, lipoma and dermoid cyst. In addition, there are reports in the literature about associations among the oral LC and other lesions such as epidermoid cyst and benign migratory glossitis [12, 17]. Costa et al. [1] reported a simultaneous occurrence of oral LC with oral squamous carcinoma cells. These cases probably represent rare associations between diseases etiologically different [17]. In the present report, there was not any association observed between the lesion and other pathologies.

The treatment of choice for oral LC is the conservative surgical excision, which has good prognosis. It has low recurrence rates and there are no reports of postoperative sensorineural complication or even malignant transformation [9, 12, 15, 17]. In the reported case, one year after biopsy, there is no evidence of recurrence. According to observations of Silva et al. [13], the probable cause of the low rates of LC recurrence is the noninfiltrative nature of the lesion. However, the patient in the present study reported clinical symptoms compatible with injury to the lingual nerve.

Heretofore, there are no reports in the literature describing the occurrence of temporary paresthesia after removal of LC in the ventral surface of the tongue. In this case, it is believed that this postoperative complication occurred possibly due to anatomical variations of the lingual nerve. Anatomically the lingual nerve emerges from the posterior trunk of the mandibular nerve and is responsible for the sensory innervation of the mouth floor mucosa, the gums in the lingual surface of the lower teeth, and the anterior two-thirds of the tongue. After passing through the infratemporal fossa, the lingual nerve enters in the tongue by the lateral surface of the hyoglossal muscle, and the lingual nerve branches are directed to the tip of the tongue, supplying afferent nerve fibers to the ventral surface of the tongue [18].

According to Rusu et al. [19], two morphological types of the lingual nerve terminal division were observed: single primary nerve trunk and two primary nerve trunks (one medial, distributed in the middle third of the tongue, and another lateral, distributed in the anterior third of the tongue). One study with cadavers [16] emphasizes the importance of the medial branch of the lingual nerve and describes five patterns of innervation in ventral surface of the tongue: 1a (the medial branch of the lingual nerve in straight direction along a reference line on the styloglossus muscle), 1b (the medial branch of the lingual nerve in straight direction along a reference line on the styloglossus muscle, with the emission of a direct branch which innervates the tongue mucosa), 2a (the medial branch of the lingual nerve that is curved along a reference line on the styloglossus muscle), 2b (the medial branch of the lingual nerve that is curved along a reference line on the styloglossus muscle, with the emission of a branch that directly innervates the lingual mucosa), and 3 (the medial branch of the lingual nerve that is anteriorly directed on the styloglossus muscle, rising vertically near the midline). It was not possible to determine exactly which nerve structure was traumatized in the surgical procedure performed in this case. However, the authors of this study believe that the occurrence of lingual paresthesia was associated with an anatomical pattern of types 1b or 2b, which have nervous branch directly innervating the lingual mucosa in the ventral surface of the tongue. In addition, the conservative surgical procedure with a superficial surgical excision in mucosa of the ventral surface of the tongue reinforces this hypothesis.

Lingual nerve injury is not very common in surgical procedures in dentistry. The lingual nerve can be damaged as a result of direct or indirect forces. Due to the anatomical location of the nerve, direct trauma to the lingual nerve may occur during various surgical procedures, for example, those carried out for the management of trauma, cysts, tumors, and preprosthetic problems, orthognathic surgery, damage caused by the use of instruments, and, most commonly, removal of the third molars. Indirect injury to the nerves can be also a result of physiological phenomena, including pressure from bruises and postsurgical edema. The overall risk of lingual nerve injury associated with third molar removal ranges from 0.2% (permanent disturbance) to 22% (sensory disturbances in the early postoperative period). The reported rate of permanent lingual nerve injury is generally in the range of 0–2% [20, 21]. The case reported in this paper is even more rare, since there is relatively minor surgical manipulation.

The administration of anesthesia may also be directly connected to the lingual nerve injury. Various mechanisms have been proposed to explain lingual nerve injury during the administration of anesthesia with an inferior alveolar nerve block injection. These include the following: neurovascular bundle damage by the penetration of a sharp needle tip, movement of the needle itself, extra- or intraneural hemorrhage from trauma to blood vessels, or the neurotoxicity of the used anesthetic site, in particular articaine 4% and prilocaine 3% to 4% [22, 23]. Because of the large variability in the anatomic position and the width of the lingual nerve in relation to the needle, little can be done to predict or prevent nerve injury [24]. Harn and Durham [25] reported 3.62% of chance to traumatize the nerve with each lingual mandibular conventional block injection administered, with a 14.99% of chance to postinjection complication, which may include neural apraxia, axonotmesis, or neurotmesis. However, the authors of this study believe that the occurrence of lingual paresthesia may be associated with the administration of anesthesia.

The majority of injuries result in transient sensory disturbances, but in some cases permanent paresthesia (abnormal sensation), hypoesthesia (reduced sensation), or, even worse, some form of dysesthesia (unpleasant abnormal sensation) can occur. Sensory loss lasting longer than 6 months is mostly permanent. The subsequent distorted sensory sensation can result in significant impairment in speech and chewing and taste loss from the ipsilateral anterior segment of the tongue, which has a negative impact on socializing and the patient's psychological well-being [20]. Other observed clinical signs include drooling, tongue biting, a burning sensation of the tongue, anesthesia resulting in burns during eating and drinking, pain, change in speech pattern, and a change in taste perception of food and drink. Dysgeusia and hypogeusia have been reported as a result of the proximity of the chorda tympani to the lingual nerve within the sheath nerve [22, 23].

Techniques of sensory assessment of the trigeminal nerve are controversial. A complete evaluation of the referred postoperative sensorial disturbance is necessary to establish the nature of the problem and to standardize the tests to follow the patient over time and to determine eventual improvement. For this purpose, commonly used sensory tests include light touch, brush directional touch, 2-point discrimination, temperature change, and pinprick. Therefore, several objective tests, such as electrophysiological tests, have been proposed and developed to avoid the possibility of bias. Some of these tests are based on nerve conduction, somatosensory evoked potentials, or reflex recordings [20, 22, 23].

The treatment of nerve injuries may be surgical or treated conservatively. As for microneurosurgical repair, complete anesthesia beyond 3 months, profound hypoesthesia with no improvement beyond 4 months, dysesthesia beyond 4 months, and clinically observed nerve severance could be indications for surgical intervention. Reconstruction of the lingual nerve is contraindicated when one of the following conditions is present: improving return of sensation, sensory deficit acceptable to the patient, central neuropathic pain, dysesthesia not resolved by a local anesthesia nerve block, medical neuropathy, medically compromised patient, or excessive delay following injury [20].

Regarding drug therapy for paresthesia treatment, a commonly established conduct is the prescription of vitamins, especially the B-complex vitamins (B1 and B12 vitamins). B-complex vitamins administration plays an important role in the conduction of nerve impulses and in the aerobic metabolism, acting in the proper functioning of the nerve system [26]. Although some authors totally discredit the effectiveness of the drug treatment, drugs currently available as the best ones seem to produce only partial relief of symptoms. Tricyclic antidepressants and carbamazepine have been shown to be helpful in some cases, although they may be associated with different side effects [20]. The use of low power lasers (LLLT—Low Level Laser Therapy) has been cited in the literature, in the dental field presenting biomodulator effect and it had been indicated in cases of painful symptoms, and tissue repair [27]. They help to reduce the production of inflammatory mediators of arachidonic acid and promote regeneration after nerve injury [28]. The irradiation by low power laser on the path of affected nerve has proven to be effective in the sensory improvement having advantage because of the absence of pain or trauma promoting greater comfort to patient [26].

The lymphoepithelial cyst is a rare lesion in the oral cavity that can be surgically treated. Although previous reports have no described tongue sensorineural disorders associated with this lesion, the occurrence of this event may be related to an unexpected anatomical variation of the lingual nerve.
